# Quantitative Analysis of Viral Load per Haploid Genome Revealed the Different Biological Features of Merkel Cell Polyomavirus Infection in Skin Tumor

**DOI:** 10.1371/journal.pone.0039954

**Published:** 2012-06-29

**Authors:** Satoshi Ota, Shumpei Ishikawa, Yutaka Takazawa, Akiteru Goto, Takeshi Fujii, Ken-ichi Ohashi, Masashi Fukayama

**Affiliations:** 1 Department of Pathology, Chiba University Hospital, University of Chiba, Chuo, Chiba, Chiba, Japan; 2 Department of Pathology, Graduate School of Medicine, University of Tokyo, Bunkyo, Tokyo, Japan; 3 Department of Pathology, Toranomon Hospital, Minato, Tokyo, Japan; Ohio State University Medical Center, United States of America

## Abstract

Merkel cell polyomavirus (MCPyV) has recently been identified in Merkel cell carcinoma (MCC), an aggressive cancer that occurs in sun-exposed skin. Conventional technologies, such as polymerase chain reaction (PCR) and immunohistochemistry, have produced conflicting results for MCPyV infections in non-MCC tumors. Therefore, we performed quantitative analyses of the MCPyV copy number in various skin tumor tissues, including MCC (n = 9) and other sun exposure-related skin tumors (basal cell carcinoma [BCC, n = 45], actinic keratosis [AK, n = 52], Bowen’s disease [n = 34], seborrheic keratosis [n = 5], primary cutaneous anaplastic large-cell lymphoma [n = 5], malignant melanoma [n = 5], and melanocytic nevus [n = 6]). In a conventional PCR analysis, MCPyV DNA was detected in MCC (9 cases; 100%), BCC (1 case; 2%), and AK (3 cases; 6%). We then used digital PCR technology to estimate the absolute viral copy number per haploid human genome in these tissues. The viral copy number per haploid genome was estimated to be around 1 in most MCC tissues, and there were marked differences between the MCC (0.119–42.8) and AK (0.02–0.07) groups. PCR-positive BCC tissue showed a similar viral load as MCC tissue (0.662). Immunohistochemistry with a monoclonal antibody against the MCPyV T antigen (CM2B4) demonstrated positive nuclear localization in most of the high-viral-load tumor groups (8 of 9 MCC and 1 BCC), but not in the low-viral-load or PCR-negative tumor groups. These results demonstrated that MCPyV infection is possibly involved in a minority of sun-exposed skin tumors, including BCC and AK, and that these tumors display different modes of infection.

## Introduction

Merkel cell carcinoma (MCC), which is a rare and aggressive primary cutaneous neoplasm that affects elderly and/or immunocompromised individuals, tends to occur in sun-exposed skin [1]. The Merkel cell polyomavirus (MCPyV) was recently identified in MCC [2], and its frequency in MCC has been reported to be 100% by immunohistochemical and/or polymerase chain reaction (PCR) studies that were performed in western countries [2]–[23] and in East Asia [24]–[27]. The monoclonal integration of MCPyV DNA in host DNA has been demonstrated in neoplastic MCC cells, indicating that the virus causes and/or promotes this specific type of cutaneous neoplasm [2]. However, it remains unclear how often MCPyV is associated with other cutaneous neoplasms and to what extent racial factors influence the infection rates. In skin tumors other than MCC, MCPyV has been detected at various frequencies (0%–25%) by PCR. However, immunohistochemical analyses have suggested that MCPyV is specific to MCC and is absent from other skin tumors, including squamous cell carcinoma, basal cell carcinoma (BCC), and lymphoma [28], [29]. MCPyV T-antigen expression may be suppressed in infected cells in certain circumstances, even though MCPyV viral DNA is integrated into the cellular DNA. A significant number of MCPyV-positive cases are positive for the small-T (ST) antigen but do not express the large-T (LT) antigen [30]. Recently, Neumann et al. found that all integrated genomes had truncation mutations in the LT antigen [31]. However, it may be difficult to address these issues without a sensitive quantitative detection method.

In the present study, we investigated the frequency of MCPyV infection in skin tumors, including MCC and other sun exposure-related skin tumors, such as BCC, actinic keratosis (AK), and Bowen’s disease (BD), in Japan. Other representative non-melanocytic, melanocytic, and lymphoid skin tumors were also included. We applied digital PCR in order to calculate the absolute viral copy number per haploid human genome [32], [33]. This method uses nanofluidic technology to randomly distribute applied DNA molecules to multiple small reaction chambers at a concentration of 0 to 1 DNA molecules per chamber. Target and reference genes are simultaneously PCR-amplified with a dual-color amplification reaction, and their copy numbers are then calculated by counting the numbers of signal-positive chambers. This PCR-efficiency-independent method is highly robust for comparing copy numbers using different primer sets. The results we obtained for viral load using this quantitative method revealed the different biological characteristics of MCPyV in these tumors and provided a reasonable explanation for the conflicting results obtained so far.

## Results

### Diagnosis of MCC

The diagnosis of MCC was confirmed by the presence of a perinuclear dot-like positive staining pattern for CK20 and positivity for chromogranin A and synaptophysin (Table 1). None of the other tumors, including a MCPyV-positive BCC tumor, displayed the same staining pattern.

**Table 1:** Clinicopathological data of Merkel cell polyomavirus (MCPyV)-positive skin tumors.

	Case	Age/sex	Tumorsize	Clinical courseand follow up	Immunocompromisedor not	Immunohistochemistry		
						CK20	ChromograninA	Synaptophysin
MCC	1	71/F	2.1×2.0×1.8 cm	No recurrence ormetastasis at 2 years	No	dot,30%	weak,100%	–
	2	62/M	3.5×2.5×2.5 cm	Primary tumor foundafter 2 months post living-donor liver transplantation.Lymph node metastasisat 6 months. Death at18 months with MCC.	Yes	dot,100%	weak,100%	weak,100%
	3	73/M	7.0×5.6×1.2 cm	Primary buttock MCCwith multiple inguinaland pelvic lymph nodemetastases. Death at6 months with MCC.	No	dot &cytoplasmic,90%	100%	weak,10%
	4	73/F	1.4×0.9×0.2 cm	No recurrence ormetastasis at 14 months.	No	dot, 90%	60%	100%
	5	59/F	0.9 cm	No recurrence ormetastasis at 70 months.	No	dot &cytoplasmic,80%	100%	100%
	6	77/M	2.7×2.6×1.0 cm	No recurrence or metastasisat 22 months. Lost tofollow up.	No	dot,100%	100%	100%
	7	76/F	5.4×3.5 cm	Multiple liver metastasesafter 2 months. Deathat 3 months	No	dot,90%	50%	90%
	8	79/F	2.4×2.2×1.8 cm	Multiple skin metastasesafter 10 months. Systemicmetastases at 12 months.Lost to follow up.	No	dot,90%	10%	100%
	9	92/F	4.1×2.5×2.5 cm	Multiple lymph nodemetastases after 6 months.Lost to follow up.	No	dot,60%	20%	100%
BCC	1	80/F	0.4×0.3 cm	No recurrence ormetastasis.	No	–	–	–
AK	1	83/F	1.0×1.0 cm		No	–	–	–
	2	63/M	1.1×1.0 cm		No	–	–	–
	3	79/F	0.8×0.6 cm		No	–	–	–

MCC, Merkel cell carcinoma; BCC, Basal cell carcinoma; AK, Actinic keratosis; CK20, Cytokeratin20.

In our MCC series, none of the MCC patients were immunocompromised, except for Case 2 in which primary MCC had developed within 2 months after a living donor liver transplantation for fulminant hepatitis of unknown etiology. The patient passed away after 18 months because of MCC recurrence and metastasis. Cases 1, 4, 5, and 6 involved limited disease without metastasis or recurrence, while Cases 2, 3, 7, 8, and 9 involved synchronous or metachronous metastases.

### PCR Amplification of MCPyV from Skin Tumors

**Figure 1:**
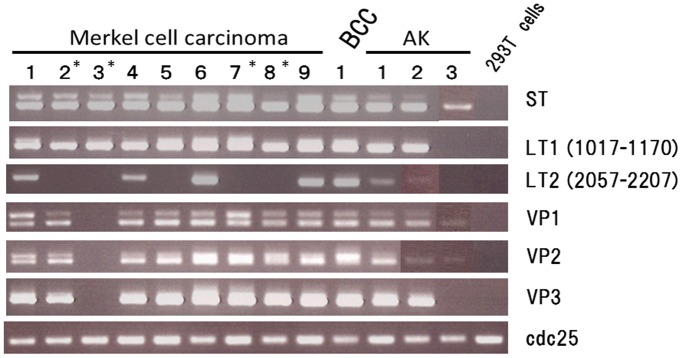
Polymerase chain reaction (PCR) amplification of the Merkel cell polyomavirus in the skin tumors. Six MCPyV gene fragments were detected in Merkel cell carcinoma, basal cell carcinoma (BCC), and actinic keratosis (AK). Cases involving synchronous or metachronous metastases are marked with an asterisk. Specific PCR fragments, including large T (LT)2, VP1, and VP2, were not amplified constantly in AK cases 2 and 3 (see text). To clarify, we replaced this part with a picture of successful amplification in another trial. Abbreviations: BCC, basal cell carcinoma; AK, actinic keratosis; 293T, polyomavirus SV40 T antigen-positive 293 cells. The lower panel indicates the single PCR proliferation band of the CDC25 gene.

We first analyzed whether MCPyV DNA fragments were present or absent in skin tumor tissues by conventional PCR. Nested PCR was performed in order to detect the 6 MCPyV DNA fragments using DNA samples extracted from tissue samples. The results are presented in Fig. 1 and Table 2. Positive results were obtained in all 9 MCC cases (100%), in 1 of 46 BCC cases (2.2%), and in 3 of 52 AK cases (5.8%). No PCR amplification fragments were observed in any of the other skin tumors, such as BD (n = 34), seborrheic keratosis (SK; n = 5), primary cutaneous anaplastic large-cell lymphoma (PCALCL; n = 5), malignant melanoma (MM; n = 5), or melanocytic nevus (MN; n = 6). Among the 6 fragments examined, the ST and LT1 fragments were amplified in 13 and 12 cases, respectively, while LT2 was the fragment that was most frequently absent from the tumors (it was only observed in 6 cases). As a result, all 6 fragments were amplified in 7 cases (4 of 9 MCC, 1 BCC, and 2 of 3 AK). In MCC, all 6 MCPyV fragments were detected in cases involving limited disease without distant metastases (Cases 1, 4, and 6), while 1 or more of the fragments was absent in 5 cases, 4 of which involved synchronous or metachronous metastases (Cases 2, 3, 7, and 8). The amplification pattern was the same in the primary and metastatic tumors in Cases 2 and 3, but an additional loss of amplification was observed in 1 of the 2 metastases in Case 8. PCR amplifications were unstable in AK cases 2 and 3 where we observed significant gel bands 2 to 4 times in 5 to 6 trials of the ST, VP1, and VP2 assays.

**Table 2:** Polymerase chain reaction, immunohistochemistry, and viral copy number per haploid human genome of MCPyV-positive skin tumors.

	Case		ST	LT1	LT2	VP1	VP2	VP3	IHC (MCPyV)	IHC staining pattern	viral CN per haploid human genome	Tumor ratio	RASSF1A hypermethylation
MCC	1	Tumor	+	+	+	+	+	+	+(30%)	heterogeneous partial	42.843	4	U
	2	Tumor	+	+	−	+	+	+	+(90%)	strong diffuse	0.369	4	M/U
		MLNM	+	+	−	+	+	+					
	3	Tumor	+	+	−	−	−	−	+(90%)	weak diffuse	1.361	4	M/U
		MLNM	+	+	−	−	−	−					
	4	Tumor	+	+	+	+	+	+	+(100%)	heterogeneous diffuse		1	M/U
	5	Tumor	+	+	−	+	+	+	−	−	0.119	2	M/U
	6	Tumor	+	+	+	+	+	+	+(80%)	heterogeneous diffuse	1.253	4	U
	7	Tumor	+	+	−	+	+	+	+(90%)	heterogeneous diffuse	1.065	4	U
	8	Tumor	+	+	−	+	+	+	+(100%)	strong diffuse	0.759	4	M/U
		Skin metastasis	+	+	−	+	+	+					
		Skin metastasis	+	+	−	−	−	+					
	9	Tumor	+	+	+	+	+	+	+(100%)	strong diffuse	0.756	4	M/U
BCC	1	Tumor	+	+	+	+	+	+	+(100%)	strong diffuse	0.662	2	M/U
AK	1	Tumor	+	+	+	+	+	+	−	−	0.068	2	U
	2	Tumor	+	+	+	+	+	+	−	−	0.031	2	U
	3	Tumor	+	−	−	+	+	−	−	−	0.019	2	U

IHC, immunohistochemistry; MCC, Merkel cell carcinoma; BCC, Basal cell carcinoma; AK, Actinic keratosis; MLNM, Multiple lymph node metastases; ST, small T; LT, large T; CN, copy number, Tumor ratio: 1, <10%; 2, >10% and <30%; 3, >30% and <70%; 4, >70%.

All PCR fragments in positive MCC, BCC, and AK cases were subjected to DNA sequencing and confirmed to belong to the MCPyV sequence. The full-length T-antigen sequence of MCPyV from the BCC case was not determined because of the small amount of available DNA.

### Immunohistochemical Analysis of the MCPyV T Antigen in Skin Tumors

**Figure 2:**
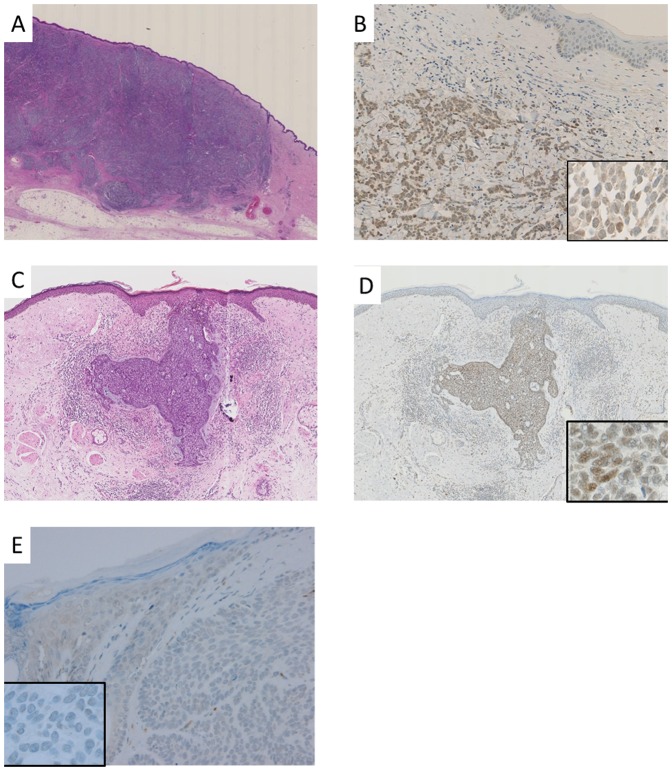
Morphology and immunohistochemical staining. Representative cases of Merkel cell carcinoma (MCC; A, B), a basal cell carcinoma (BCC)-positive case (C, D), and a BCC-negative case (E). Immunohistochemical staining with the anti-MCPyV large T-antigen antibody (CM2B4) (B, D, E). Heterogeneous and diffuse staining was observed in MCC (B), and strong diffuse positivity (D) and total negativity (E) was detected in BCC. Inset: Nuclear staining of MCPyV in MCC (B) and BCC (D,E).

Immunohistochemical analyses of MCPyV were performed to determine the cellular localization and histological distribution of the virus in tumor tissues. Full-section skin-tumor slides were immunohistochemically analyzed with an antibody (CM2B4) against the MCPyV T antigen (Fig. 2). Most MCC cases (8/9) and 1 BCC case (1/46) were positive for the MCPyV T antigen, and they all were also found to be positive in the PCR analysis (Table 2). A diffuse nuclear staining pattern was observed in most of the positive cases. The labeling ratio ranged from 80% to 100%, except for in 1 case (Case 1, 30%). The staining intensity of the tumor cell nuclei was strong in 4 cases, including the BCC case, while it was diffusely weak and/or heterogeneous in the other cases. In contrast to the positive PCR results, no positive staining was observed in AK tumors. No immunoreactivity for the MCPyV T antigen was detected in BD (n = 34), SK (n = 5), PCALCL (n = 5), MM (n = 5), or MN (n = 6) tissues, and these results were consistent with the PCR results.

### Copy Number of MCPyV in Skin Tumors

**Figure 3:**
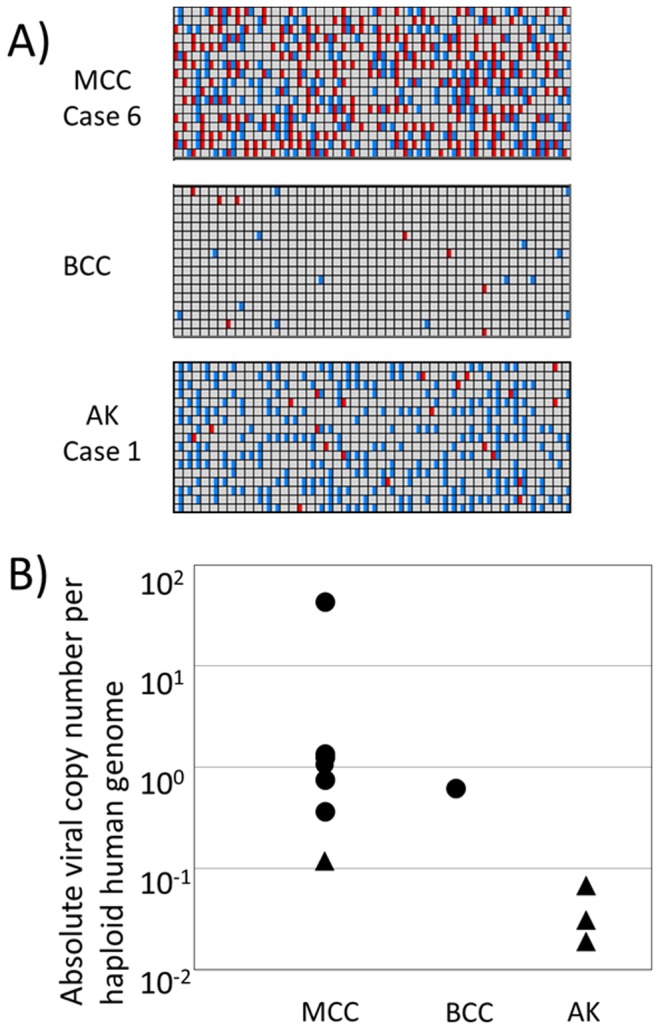
MCPyV copy number in various skin tumors. (A) Digital PCR software-generated composite heat maps showing chambers with positive signals for both control RNaseP genes (blue) and MCPyV (red). Digital PCR heat maps are indicated in the upper panel for Merkel cell carcinoma case 6, in the middle panel for basal cell carcinoma (positive case), and in the lower panel for actinic keratosis (case 1). (B) Scatter plot of the MCPyV copy number of Merkel cell carcinoma, basal cell carcinoma, and actinic keratosis. Immunohistochemically positive cases are shown as black dots (▪), and negative cases are indicated by triangles (▴).

In order to further investigate the mode of infection and discrepancies between the PCR and immunohistochemistry results, we performed digital-PCR-based quantitation of the absolute viral copy number per human genome in MCPyV-infected tumor tissue. Digital PCR analyses were performed using a DNA template that was extracted from full-section slides. Case 4 was excluded from the digital PCR analysis because its tumor cell ratio was very low (approximately 3%). We designed a MCPyV-specific primer set that targeted the ST region because this fragment was amplified in all infected cases in the present study (Fig. 1 and Table 1). The ST region overlaps with the target regions of the LT3 primer sets that were used in previous studies [8], [29], [34]. In order to avoid possible assay errors due to MCPyV sequence diversity, we confirmed the digital PCR results with an additional second primer set and found that those results were reproducible (data not shown). As a human genome reference, we used the RNaseP gene, a single copy of which exists per human haploid genome [32], [33]. We performed a dual-color assay and used the results to calculate the absolute viral copy number per haploid human genome (Fig. 3). In MCC, the tissue viral load varied from 0.119 to 42.843 (copies/haploid genome), but was mostly distributed around 1 (Fig. 3 and Table 2). The viral load was generally lower by 1 order of magnitude in AK tissue (between 0.019 and 0.068). The negative immunohistochemical results for 1 MCC and 3 AK cases were clearly linked to their low viral loads. The viral load of MCPyV-positive BCC was more similar to that of MCC tumors (0.662).

### Methylation Status of Skin Tumors

The epigenetic silencing of tumor suppressor genes, such as the RASSF1A promoter, plays a characteristic and essential role in cancer development. Host RASSF1A DNA hypermethylation has been demonstrated in SV40 polyomavirus-related tumors and cell lines and in some cases of MCC [35], [36]. Thus, our skin tumor samples were subjected to methylation-specific PCR analyses.

RASSF1A hypermethylation was detected in 6 of 9 MCC cases (67%), 7 of 46 BCC cases (15%), and 1 of 52 AK cases (1.9%) (Table 2). Interestingly, RASSF1A promoter hypermethylation was also observed in MCPyV-positive BCC. No promoter hypermethylation was seen in any other of the following skin tumors: PCALCL (n = 5), MM (n = 5), MN (n = 6), SK (n = 5), or BD (n = 34). No promoter hypermethylation of FHIT or CDKN2A was identified in MCC, BCC, AK, or other skin tumors (data not shown).

## Discussion

In the present study, the frequency of MCPyV infection in various skin tumors was analyzed by conventional PCR and immunohistochemistry, and digital PCR technology was applied to calculate the absolute viral copy number per haploid genome in these tumor tissues.

The 100% PCR-based MCPyV detection rate that was observed in MCC in this study was compatible with the findings of studies performed in the US and Europe, but it was somewhat higher than those reported in Australia and Japan [2]–[5], [9], [18], [24]. The MCPyV detection rates in 2 reports from Europe [10] and Asia [26] were over 90%, and this was similar to our detection rate. One of the reasons for our 100% positive PCR results may be due to a simple sampling problem because of the limited number of cases, and another possible reason was MCPyV sequence polymorphism within primer design regions. In the present study, the nested primer sets targeting 6 different regions of MCPyV were adopted for viral detection [24]. Interestingly, loss of the LT2 fragment was frequently observed in metastatic MCC and in primary MCC that produced metastases. While all 6 MCPyV fragments were amplified in 3 of the 4 cases involving limited disease, the LT2 fragment was absent from 4 of the 5 cases involving synchronous or metachronous metastases. While it could be due to sequence diversity in these regions, it is possible that extensive somatic mutations or deletions in these regions could be associated with tumor progression. A previous study found that a mutation in the LT region produced oncogenic effects through a prematurely truncated LT protein [30], [37]. Similar events have been demonstrated to be involved in the transformation process in animal polyomavirus models [38]–[41].

The presence and pathogenesis of MCPyV DNA in skin tumors other than MCC are controversial. In previous studies, MCPyV DNA was amplified by PCR from 32% of sporadic non-melanoma skin cancers, including BCC (36/96, 37.5% and 3/24, 12.5%), SCC (7/28, 25%), and BD (4/23, 17.4%) [4], [42]. In contrast, an immunohistochemical study did not detect any positive BCC or SCC cases [28]. The major problem with these previous studies was the lack of a method for quantitatively assessing viral infection. Conventional PCR can amplify very small amounts of viral DNA and provide us with the same positive results in spite of different viral loads, whereas the immunohistochemical method is dependent on the level of protein expression and it is difficult to reliably detect low levels of proteins. In the present study, we used digital PCR technology to calculate the absolute viral load per haploid human genome. The nanofluidic-based physical separation of each DNA template makes this technology highly robust, despite differences in the PCR efficiencies of different primers, such as RNaseP and MCPyV ST. Assessing the absolute viral load per haploid human genome is highly informative. First, the viral load differed markedly between MCC (0.37–42.8) and AK (0.02–0.07), suggesting that the biology of MCPyV infection differs between these 2 tumor groups. Second, there was a strong correlation between the immunohistochemical findings and viral load, which explains the conflicting results that were obtained with conventional PCR and immunohistochemistry. One possibility is that MCPyV-containing lymphocytes infiltrate within or around the atypical epidermis in AK. Another possibility is the infection of a small subset of tumor cells. It is worth noting that a lack of immunostaining and a relatively low copy number were observed in 1 MCC case (0.119 in Case 5). Therefore, we could not rule out the possibility that MCPyV had infected AK cells in our AK cases, and further studies are needed to examine this. Third, in most MCC cases, the MCPyV copy number per haploid genome was around 1. Taking the diffuse immunohistochemical staining seen in the majority of MCC cells into account, there is a realistic possibility that each MCC cell had clonally integrated 2 copies of the MCPyV genome, which could not be the case for AK.

In the present study, we observed the presence of MCPyV DNA fragments in 1 of 46 BCC cases (2.2%). The strong and diffusely positive immunohistochemical staining and moderate viral load (compared to that observed in the MCC) observed in this tumor confirmed that it had been infected by MCPyV. These findings suggest that MCPyV may also contribute to the development of the minority of sun-exposed skin tumors in addition to MCC. Interestingly, hypermethylation of RASSF1A was detected in this case of BCC, as was found in two-thirds of the MCC cases. Hypermethylation of host DNA has been detected in SV40 polyomavirus-related tumors and cell lines as well as in some MCC [13], [14]. MCPyV infects progenitor skin endocrine cells, but it may sometimes infect cells that can differentiate into other cell types.

Although further studies are needed for a complete understanding of these results, our quantitative analysis of the viral load per haploid genome revealed that MCPyV infection displays different biological characteristics and epidemiology in skin tumor tissues.

## Materials and Methods

### Tissue and Cell Samples

Skin tumors, which were surgically resected or biopsied from 1996 to 2009, were retrieved from the database of the Department of Pathology, Tokyo University Hospital. Each histological diagnosis was independently confirmed by S.O and Y.T. Skin tumors used in this study included MCC (n = 4), BCC (n = 46), AK (n = 52), BD (n = 34), SK (n = 5), PCALCL (n = 5), MM (n = 5), and MN (n = 6). Additionally, 5 cases of MCC from Toranomon Hospital were also analyzed. All of these tumors were fixed by formalin and embedded in paraffin for diagnostic purposes. Immunostaining of CK20, synaptophysin, and chromogranin A was used to confirm the diagnosis of MCC. This study was approved by the University of Tokyo Institutional Ethical Committee. Clinical samples with written informed consent were collected under the University of Tokyo Institutional guidelines for the study of human tissues.

As for the cultured cells, 293T cells (American Type Culture Collection, Manassas, VA) were maintained, as described previously.

### Preparation of DNA from Paraffin-embedded Clinical Material

Serial sections of tumor specimens were subjected to hematoxylin and eosin staining, immunohistochemistry, and DNA preparation. To isolate DNA from formalin-fixed paraffin-embedded skin tumor samples, 3 10 µm-thick sections were placed into 1.5-mL sterile tubes, and a DNeasy Tissue Kit (QIAGEN GmbH, Hilden, Germany) was used to purify DNA according to the manufacturer’s instructions. Extracted DNA was used for PCR and digital PCR.

### PCR Primers for Polyomavirus DNA

The quality of DNA was checked by amplifying the cdc25 (forward: 5′-TGGTGGGCCAAACACTATCC-3′, reverse: 5′-ATCGTTGGGCTCGCAGATCACC-3′) and glyceraldehyde-3-phosphate dehydrogenase (forward: 5′-GAAGGTGAAGGTCGGAGTC-3′, reverse: 5′-GAAGATGGTGATGGGATTC-3′) genes.

For MCPyV detection, 6 nested primer sets, including primers for ST, LT, and VP1-3 regions were prepared, and nested PCR was performed, as described previously [24], with 40 ng of extracted DNA.

### DNA Sequencing

PCR-amplified fragments of MCPyV and other polyomaviruses were purified using MicroSpin S-300 HR Columns (GE Healthcare, Piscataway, NJ), and purified PCR products were then applied to an ABI sequencer (Life Technologies Corporation, Carlsbad, CA) and analyzed according to the manufacturer’s protocol. All sequences of PCR-amplified fragments were compared to each other for similarity using NCBI-BLAST and were fully matched with the Merkel cell polyomavirus genome sequence, which was already reported [37]. Additional Merkel Cell Polyomavirus sequencing for hot spot in Large T antigen was analyzed in Figure S2.

### Antibodies and Immunohistochemistry

Immunohistochemistry was applied to formalin-fixed and paraffin-embedded tissue samples in all cases. Immunohistochemistry was performed with monoclonal antibodies against the MCPyV LT antigen (CM2B4; Santa Cruz Biotechnology, Inc, Santa Cruz, CA, 1∶50 dilution), CK20 (Leica Microsystems Inc, Buffalo Grove, IL, 1∶100 dilution), chromogranin A (Dako Denmark A/S, Glostrup, Denmark, 1∶200 dilution), and synaptophysin (Dako Denmark A/S, 1∶100 dilution). Immunohistochemistry was performed according to standard techniques on a Ventana Benchmarks XT Autostainer (Ventana Medical Systems, Inc, Tucson, AZ) with the labeled streptavidin-biotin peroxidase method and diaminobenzidine visualization. Appropriate positive and negative controls were included for each immunohistochemical experiment.

Nuclear staining was considered to indicate positivity for the LT antigen of MCPyV.

### Copy Number Assessment Using Digital PCR

A primer set targeting the ST region, which overlaps with the target regions of the LT3 primer sets used in previous studies, was designed (STF 576: 5′-TCGCCAGCATTGTAGTCTAAAAAC-3′; STR 668: 5′-CCAAACCAAAGAATAAAGCACTGA-3′, and ST probe: 5′-AGCAAAAACACTCTCCCCACGTCAGACA-3′) (Fig. S1). For additional digital PCR quantification, a second primer set was designed (STF 550: 5′-TGCGCTTGTATTAGCTGTAAGTTGT-3′; STR 640: 5′-AAAACACTCTCCCCACGTCAGA-3′; and ST probe: 5′-AGCAAAAACACTCTCCCCACGTCAGACA-3′).

For each panel, 10 µL of reaction mixture containing 1 × TaqMan Gene Expression Master Mix (Life Technologies), 1 × RNase P-VIC TaqMan assay, 1 × MCPyV ST-FAM TaqMan assay (900 nM primers and 200 nM probe), 1 × sample loading reagent (Fluidigm Corporation, South San Francisco, CA), and 3.5 µL of extracted genomic DNA was prepared. The reaction mix was applied to the 12.765 digital array, which contained 765 small chambers for each sample, and was analyzed using the EP-1 system (Fluidigm Corporation) [33]. Thermocycling conditions included an initial step of 95°C for 10 min, which was followed by 40 cycles of 2-step PCR: 15 s at 95°C for denaturing and 1 min at 60°C for annealing and extension. Data was transformed from the observed positive chamber count to the estimated copy number using the mathematical formula described by Dube S et al. [32], and the absolute viral copy number per haploid genome was defined as the ratio of MCPyV ST copy number to RNaseP copy number. Tumor cell ratios were counted and graded as follows: 1, <10%; 2, >10% and <30%; 3, >30% and <70%; or 4, >70%. The absolute viral copy number per haploid genome by the second primer showed similar results (data not shown).

### Methylation-specific PCR (MS-PCR)

Methylation analysis was performed to evaluate the promoter hypermethylation status of MCC, BCC, and AK. The promoter regions of RASSF1A, CDKN2A, and FHIT were examined, as described previously [10]. The extracted template DNA was modified by the bisulfite reaction using an EpiTect Bisulfite kit (QIAGEN GmbH). Methylation status was distinguished by MS-PCR using sequence-specific primer pairs. MS-PCR experiments were performed at least twice. PCR primers and conditions were described previously [10].

## Supporting Information

Figure S1 **The primer used for digital PCR targeting the ST region, which overlaps with the target regions of the LT3 primer that was previously reported by Feng.**
(DOCX)

Figure S2 **Merkel Cell Polyomavirus sequencing for hot spot in Large T antigen.**
(DOC)
