# Supplementary material for: Quantitative Analysis of Viral Load per Haploid Genome Revealed the Different Biological Features of Merkel Cell Polyomavirus Infection in Skin Tumor
Source: PLoS One. 2012 Jun 29;7(6):e39954. doi: 10.1371/journal.pone.0039954 (PMC3386999; doi:10.1371/journal.pone.0039954)
Supplement: Figure S1 — The primer used for digital PCR targeting the ST region, which overlaps with the target regions of the LT3 primer that was previously reported by Feng. (DOCX) [file pone.0039954.s001.docx]

Figure S1

LT3 forward:F STF 576(digital PCR primer: F)

571 **ttgtctcgcc agcattgtag tctaaaaac**t ttaaagcaaa aaaactgtct gacgtgggga gagtgttttt gctatcagtg ctttattctt tggtttggat ttcctcctac

aacagagcgg tcgtaacatc agatttttga aatttcgttt ttttg**acaga ctgcacccct ctcacaaaaa cga**t**agtcac gaaataagaa accaaacc**ta aaggaggatg

ST probe(digital PCR primer: R) STR 668(digital PCR primer: R)

681 ttgggaaagt tttgactggt ggcaaaaaac tttagaagaa actgactact gcttactgca tctgcacctt ttctagactc ctacttcctt cctctgtaag tattagatat

aaccctttca aaactgacca ccgttttttg aaatcttctt tgactgatga cgaatgacgt agacgtggaa aagatctgag gatgaaggaa ggagacattc ataatctata

791 ggaaaagtct ataaggcaaa atatcaaaga aaggttattt atgacagatt ttctgtactt tcccatctag gttgacgagg cccctatata tgggaccact aaattcaaag

ccttttcaga tattccgttt tatagtttct ttccaataaa tactgtctaa aagacatgaa agggtagat**c caactgctcc ggggatata**t accctggtga tttaagtttc

**LT3 reverse**
