# Supplementary material for: Quantitative Analysis of Viral Load per Haploid Genome Revealed the Different Biological Features of Merkel Cell Polyomavirus Infection in Skin Tumor
Source: PLoS One. 2012 Jun 29;7(6):e39954. doi: 10.1371/journal.pone.0039954 (PMC3386999; doi:10.1371/journal.pone.0039954)
Supplement: Figure S2 — Merkel Cell Polyomavirus sequencing for hot spot in Large T antigen. (DOC) [file pone.0039954.s002.doc]

**Figure S2**

**Merkel Cell Polyomavirus sequencing for hot spot in Large T antigen**

**A,T,G,C: Mutation site,**

**Forward primer and Reverse primer: Primer designation site to check MCPyV DNA sequence**

| Primers Used for partial MCPyV Sequence were designated under the the GenBank  database of MCC350 sequence, EU375803 | | | |
| --- | --- | --- | --- |
| Pimer set | F/R | Position | 5'–3' |
| A | Forward | 1374-1394 | GAGGCCTCGTCATCTCAGTT |
|  | Reverse | 1603-1579 | GCATGGCTAAGATAATCAGAAACA |
|  |  |  |  |
| B | Forward | 1711-1730 | GGCATGCCTGTGAATTAGGA |
|  | Reverse | 1905-1885 | GCAGTGGCTTATTCTCTTGC |
|  |  |  |  |
| C | Forward | 1868-1887 | GCCCCCTTACAAATTACTGC |
|  | Reverse | 2055-2034 | CACTTTTGGCAAGGAAATGG |
|  |  |  |  |
| D | Forward | 2062-2081 | GATCTCGCCTCAAACCTCAC |
|  | Reverse | 2210-2190 | CCTGGTCATTTCCAGCATCT |

| PCR amplification | |  | |  | |  | |  |  |  | |
| --- | --- | --- | --- | --- | --- | --- | --- | --- | --- | --- | --- |
|  | Primer set | | | | | | | | Control | | |
|  | PCR for sequencing | | | | | | | Initial PCR experiments | | | |
| Case | A | | B | | C | | D | LT2 | cdc25 | | GAPDH |
| MCC1 | ○ | | ○ | | ○ | | ○ | ○ | ○ | | ○ |
| MCC2 | × | | × | | × | | × | × | ○ | | ○ |
| MCC3 | × | | × | | × | | × | × | ○ | | ○ |
| MCC4 | ○ | | ○ | | ○ | | ○ | ○ | ○ | | ○ |
| MCC5 | × | | × | | × | | × | × | ○ | | ○ |
| MCC6 | ○ | | ○ | | ○ | | ○ | ○ | ○ | | ○ |
| MCC7 | × | | × | | × | | × | × | ○ | | ○ |
| MCC8 | × | | × | | × | | × | × | ○ | | ○ |
| MCC9 | ○ | | ○ | | ○ | | ○ | ○ | ○ | | ○ |
| BCC | - | | - | | - | | - | ○ | ○ | | ○ |
| AK1 | × | | ○ | | ○ | | ○ | ○ | ○ | | ○ |
| AK2 | × | | ○ | | ○ | | ○ | ○ | ○ | | ○ |
| AK3 | × | | ○ | | × | | × | × | ○ | | ○ |
| ○：　Successful PCR amplification, X: No PCR amplification | | | | | | | | |  | |  |
| BCC: No residual material, because the initial material was a tiny biopsy. | | | | | | | | |  | |  |

**MCC350t; FJ173805**

**MCC350; EU375803,**

**MCC366; FJ173803**

**MCC339; FJ173806**

**MCC206; FJ173812**

**PBMC85; FJ173804**

**MCC352; FJ173814**

**MCC348; FJ173809**

**MCC347; FJ173810**

**MCC322; FJ173811**

**MCC344; FJ173807**

**MCC349; FJ173813**

**MCC345; FJ173808**

**MKL-1, FJ173815**

**FJ173805: AGCCCCCGTGTTCCTCTGCCGAGGAGGCCTCGTCATCTCAGTTTACAGAT 1205**

**EU375803： AGCCCCCGTGTTCCTCTGCCGAGGAGGCCTCGTCATCTCAGTTTACAGAT 1400**

**FJ173803： AGCCCCCGTCTTCCTCTGCCGAGGAGGCCTCGTCATCTCAGTTTACAGAT 1205**

**FJ173806： AGCCCCCGTCTTCCTCTGCCGAGGAGGCCTCGTCATCTCAGTTTACAGAT 1205**

**FJ173812： AGCCCCCGTCTTCCTCTGCCGAGGAGGCCTCGTCATCTCAGTTTACAGAT 1205**

**FJ173804： AGCCCCCGTCTTCCTCTGCCGAGGAGGCCTCGTCATCTCAGTTTACAGAT 1205**

**FJ173814： AGCCCCCGTCTTCCTCTGCCGAGGAGGCCTCGTCATCTCAGTTTACAGAT 1205**

**FJ173809： AGCCCCCGTCTTCCTCTGCCGAGGAGGCCTCGTCATCTCAGTTTACAGAT 1205**

**FJ173810： AGCCCCCGTCTTCCTCTGCCGAGGAGGCCTCGTCATCTCAGTTTACAGAT 1205**

**FJ173811： AGCCCCCGTCTTCCTCTGCCGAGGAGGCCTCGTCATCTCAGTTTACAGAT 1205**

**FJ173807： AGCCCCCGTCTTCCTCTGCCGAGGAGGCCTCGTCATCTCAGTTTACAGAT 1205**

**FJ173813： AGCCCCCGTGTTCCTCTGCCGAGGAGGCCTCGTCATCTCAGTTTACAGAT 1205**

**FJ173808： AGCCCCCGTGTTCCTCTGCCGAGGAGGCCTCGTCATCTCAGTTTACAGAT 1205**

**FJ173815： AGCCCCCGTGTTCCTCTGCCGAGGAGGCCTCGTCATCTCAGTTTACAGAT 1400**

**Forward primer**

**MCC1: GAGGCCTCGTCATCTCAGTTTACAGAT 1400**

**MCC4: GAGGCCTCGTCATCTCAGTTTACAGAT 1400**

**MCC6: GAGGCCTCGTCATCTCAGTTTACAGAT 1400**

**MCC9: GAGGCCTCGTCATCTCAGTTTACAGAT 1400**

**FJ173805: TAGGAATACATATCCTCCTCCTTCACCACCCCGAAGACCCCTCCTCCATT 1255**

**EU375803： TAGGAATACATATCCTCCTCCTTCACCACCCCGAAGACCCCTCCTCCATT 1450**

**FJ173803： GAGGAATACAGATCCTCCTCCTTCACCACCCCGAAGACCCCTCCTCCATT 1255**

**FJ173806： GAGGAATACAGATCCTCCTCCTTCACCACCCCGAAGACCCCTCCTCCATT 1255**

**FJ173812： GAGGAATACAGATCCTCCTCCTTCACCACCCCGAAGACCCCTCCTCCATT 1255**

**FJ173804： GAGGAATACAGATCCTCCTCCTTCACCACCCCGAAGACCCCTCCTCCATT 1255**

**FJ173814： GAGGAATACAGATCCTCCTCCTTCACCACCCCGAAGACCCCTCCTCCATT 1255**

**FJ173809： GAGGAATACAGATCCTCCTCCTTCACCACCCCGAAGACCCCTCCTCCATT 1255**

**FJ173810： GAGGAATACAGATCCTCCTCCTTCACCACCCCGAAGACCCCTCCTCCATT 1255**

**FJ173811： GAGGAATACAGATCCTCCTCCTTCACCACCCCGAAGACCCCTCCTCCATT 1255**

**FJ173807： GAGGAATACAGATCCTCCTCCTTCACCACCCCGAAGACCCCTCCTCCATT 1255**

**FJ173813： GAGGAATACAGATCCTCCTCCTTCACCACCCCGAAGACCCCTCCTCCATT 1255**

**FJ173808： GAGGAATACAGATCCTCCTCCTTCACCACCCCGAAGACCCCTCCTCCATT 1255**

**FJ173815： GAGGAATACAGATCCTCCTCCTTCACCACCCCGAAGACCCCTCCTCCATT 1450**

**MCC1: GAGGAATACAGATCCTCCTCCTTCACCACCCCGAAGACCCCTCCTCCATT 1450**

**MCC4: GAGGAATACAGATCCTCCTCCTTCACCACCCCGAAGACCCCTCCTCCATT 1450**

**MCC6: GAGGAATGCAGATCCTCCTCCTTCACCACCCCGAAGACCCCTCCTCCATT 1450**

**MCC9: GAGGAATGCAGATCCTCCTCCTTCACCACCCCGAAGACCCCTCCTCCATT 1450**

**FJ173805: CTCAAGAAAGCGAAAATTTGGGGGGTCCCGAAGCTCTGCAAGCTCTGCTA 1305**

**EU375803： CTCAAGAAAGCGAAAATTTGGGGGGTCCCGAAGCTCTGCAAGCTCTGCTA 1500**

**FJ173803： CTCAAGAAAGCGAAAATTTGGGGGGTCCCGAAGCTCTGCAAGCTCTGCTA 1305**

**FJ173806： CTCAAGAAAGCGAAAATTTGGGGGGTCCCGAAGCTCTGCAAGCTCTGCTA 1305**

**FJ173812： CTCAAGAAAGCGAAAATTTGGGGGGTCCCGAAGCTCTGCAAGCTCTGCTA 1305**

**FJ173804： CTCAAGAAAGCGAAAATTTGGGGGGTCCCGAAGCTCTGCAAGCTCTGCTA 1305**

**FJ173814： CTCAAGAAAGCGAAAATTTGGGGGGTGCCGAAGCTCTGCAAGCTCTGCTA 1305**

**FJ173809： CTCAAGAAAGCGAAAATTTGGGGGGTCCCGAAGCTCTGCAAGCTCTGCTA 1305**

**FJ173810： CTCAAGAAAGCGAAAATTTGGGGGGTCCCGAAGCTCTGCAAGCTCTGCTA 1305**

**FJ173811： CTCAAGAAAGCGAAAATTTGGGGGGTCCCGAAGCTCTGCAAGCTCTGCTA 1305**

**FJ173807： CTGAAGAAAGCGAAAATTTGGGGGGTCCCGAAGCTCTGCAAGCTCTGCTA 1305**

**FJ173813： CTCAAGAAAGCGAAAATTTGGGGGGTCCCGAAGCTCTGCAAGCTCTGCTA 1305**

**FJ173808： CTCAAGAAAGCGAAAATTTGGGGGGTCCCGAAGCTCTGCAAGCTCTGCTA 1305**

**FJ173815： CTCAAGAAAGCGAAAATTTGGGGGGTCCCGAAGCTCTGCAAGCTCTGCTA 1500**

**MCC1: CTCAAGAAAGCGAAAATTTGGGGGGTCCCGAAGCTCTGCAAGCTCTGCTA 1500**

**MCC4: CTCAAGAAAGCGAAAATTTGGGGGGTCCCGAAGCTCTGCAAGCTCTGCTA 1500**

**MCC6: CTCAAGAAAGTGAAAATTTGGGGGGTCCCGAAGCTCTGCAAGCTCTGCTA 1500**

**MCC9: CTGAAGAAAGCGAAAATTTGGGGGGTCCCGAAGCTCTGCAAGCTCTGCTA 1500**

**Truncation mutation**

**FJ173805: GTTCAGCAAGTTTTACAAGCACTCCACCAAAGCTAAAAAACAACAGAGAA　1355**

**EU375803： GTTCAGCAAGTTTTACAAGCACTCCACCAAAGCTAAAAAACAACAGAGAA　1550**

**FJ173803： GTTCAGCAAGTTTTACAAGCACTCCACCAAAGCCAAAAAAGAACAGAGAA　1355**

**FJ173806： GTTCAGCAAGTTTTACAAGCACTCCACCAAAGCCAAAAAAGAACAGAGAA　1355**

**FJ173812： GTTCAGCAAGTTCTACAAGCACTCCACCAAAGCCAAAAAAGAACAGAGAA　1355**

**FJ173804： GTTCAGCAAGTTTTACAAGCACTCCACCAAAGCCAAAAAAGAACAGAGAA　1355**

**FJ173814： GTTGAGCAAGTTTTACAAGCACTCCACCAAAGCCAAAAAAGAACAGAGAA　1355**

**FJ173809： GTTCAGCAAGTTTTACAAGCACTCCACCAAAGCCAAAAAAGAACAGAGAA　1355**

**FJ173810： GTTCAGCAAGTTTTACAAGCACTCCACCAAAGCCAAAAAAGAACAGAGAA　1355**

**FJ173811： GTTCAGCAAGTTTTACAAGCACTCCACCAAAGCCAAAAAAGAACAGAGAA　1355**

**FJ173807： GTTCAGCAAGTTTTACAAGCACTTCACCAAAGCCAAAAAAGAACAGAGAA　1355**

**FJ173813： GTTCAGCAAGTTTTACAAGCACTCCACCAAAGCCAAAAAASAACAGAGAA　1355**

**FJ173808： GTTCAGCAAGTTTTACAAGCACTCCACCAAAGCCAAAAAAGAACAGAGAA　1355**

**FJ173815： GTTCAGCAAGTTTTACAAGCACTCCACCAAAGCCAAAAAAGAACAGAGAA　1550**

**MCC1: GTTCAGCAAGTTTTACAAGCACTCCACCAAAGCCAAAAAAGAACAGAGAA　1550**

**MCC4: GTTCAGCAAGTTTTACAAGCACTCCACCAAAGCCAAAAAAGAACAGAGAA　1550**

**MCC6: GTTCAGCAAGTTTTAGAAGTACTTCACCAAAGCCAAAAAAGAACAGAGAA　1550**

**MCC9: GTTCAGCAAGTTTTACAAGCACTCCACCAAAGCCAAAAAAGAACAGAGAA　1550**

**FJ173805: ACTCCTGTTCCTACTAATTTTCCTATTGATGTTTCTGATTATCTTAGCCA　1405**

**EU375803： ACTCCTGTTCCTACTAATTTTCCTATTGATGTTTCTGATTATCTTAGCCA　1600**

**FJ173803： ACTCCTGTTCCTACTGATTTTCCTATTGATCTTTCTGATTATCTTAGCCA　1405**

**FJ173806： ACTCCTGTTCCTACTGATTTTCCTATTGATCTTTCTGATTATCTTAGCCA　1405**

**FJ173812： ACTCCTGTTCCTACTGATTTTCCTATTGATCTTTCTGATTATCTTAGCCA　1405**

**FJ173804： ACTCCTGTTCCTACTGATTTTCCTATTGATCTTTCTGATTATCTTAGCCA　1405**

**FJ173814： ACTCCTGTTCCTACTGATTTTCCTATTGATCTTTCTGATTATCTTAGCCA　1405**

**FJ173809： ACTCCTGTTCCTACTGATTTTCCTATTGATCTTTCTGATTATCTTAGCCA　1405**

**FJ173810： ACTCCTGTTCCTACTGATTTTCCTATTGATCTTTCTGATTATCTTAGCCA　1405**

**FJ173811： ACTCCTGTTCCTACTGATTTTCCTATTGATCTTTCTGATTATCTTAGCCA　1405**

**FJ173807： ACTCCTGTTCCTACTGATTTTCCTATTGATCTTTCTGATTATCTTAGCCA　1405**

**FJ173813： ACTCCTGTTCCTACTGATTTTCCTATTGATCTTTCTGATTATCTTAGCCA　1405**

**FJ173808： ACTCCTGTTCCTACTGATTTTCCTATTGATCTTTCTGATTAT--------　1397**

**FJ173815： ACTCCTGTTCCTACTGATTTTCCTATTGATCTTTCTGATTATCTTAGCCA　1600**

**MCC1: ACTCCTGTTCCTACTGATTTTCCTATTGATGTTTCTGATTATCTTAGCCA　1600**

**MCC4: ACTCCTGTTCCTACTGATTTTCCTATTGATGTTTCTGATTATCTTAGCCA　1600**

**MCC6: ACTCCTGTTCCTACTGATTTTCCTATTGATGTTTCTGATTATCTTAGCCA　1600**

**MCC9: ACTCCTGTTCCTACTGATTTTCCTATTGATGTTTCTGATTATCTTAGCCA　1600**

**Reverse primer**

**FJ173805: TGCTGTATATAGTAATAAAACAGTAAGTTGTTTTGCCATTTATACTACTT 1455**

**EU375803： TGCTGTATATAGTAATAAAACAGTAAGTTGTTTTGCCATTTATACTACTT 1650**

**FJ173803： TGCTGTATATAGTAATAAAACAGTAAGTTGTTTTGCCATTTATACTACTT 1455**

**FJ173806： TGCTGTATATAGTAATAAAACAGTAAGTTGTTTTGCCATTTATACTACTT 1455**

**FJ173812： TGCTGTATATAGTAATAAAACAGTAAGTTGTTTTGCCATTTATACTACTT 1455**

**FJ173804： TGCTGTATATAGTAATAAAACAGTAAGTTGTTTTGCCATTTATACTACTT 1455**

**FJ173814： TGCTGTATATAGTAATAAAACAGTAAGTTGTTTTGCCATTTATACTACTT 1455**

**FJ173809： TGCTGTATATAGTAATAAAACAGTAAGTTGTTTTGCCATTTATACTACTT 1455**

**FJ173810： TGCTGTATATAGTAATAAAACAGTAAGTTGTTTTGCCATTTATACTACTT 1455**

**FJ173811： TGCTGTATATAGTAATAAAACAGTAAGTTGTTTTGCCATTTATACTACTT 1455**

**FJ173807： TGCTGTATATAGTAATAAAACAGTAAGTTGTTTTGCCATTTATACTACTT 1455**

**FJ173813： TGCTGTATATAGTAATAAAACAGTAAGTTGTTTTGCCATTTATACTACTT 1455**

**FJ173808： --------------------------------------------------**

**FJ173815： TGCTGTATATA--------------------------------------- 1611**

**MCC1: TGC**

**MCC4: TGC**

**MCC6: TGC**

**MCC9: TGC**

**FJ173805: CTGATAAAGCTATAGAGTTATATGATAAGATTGAGAAATTTAAAGTTGAT 1505**

**EU375803： CTGATAAAGCTATAGAGTTATATGATAAGATTGAGAAATTTAAAGTTGAT 1700**

**FJ173803： CTGATAAAGCTATAGAGTTATATGATAAGATTGAGAAATTTAAAGTTGAT 1505**

**FJ173806： CTGATAAAGCTATAGAGTTATATGATAAGATTGAGAAATTTAAAGTTGAT 1505**

**FJ173812： CTGATAAAGCTATAGAGTTATATGATAAGATTGAGAAATTTAAAGTTGAT 1505**

**FJ173804： CTGATAAAGCTATAGAGTTATATGATAAGATTGAGAAATTTAAAGTTGAT 1505**

**FJ173814： CTGATAAAGCTATAGAGTTATATGATAAGATTGAGAAATTTAAAGTTGAT 1505**

**FJ173809： CTGATAAAGCTATAGAGTTATATGATAAGATTGAGAAATTTAAAGTTGAT 1505**

**FJ173810： CTGATAAAGCTATAGAGTTATATGATAAGATTGAGAAATTTAAAGTTGAT 1505**

**FJ173811： CTGATAAAGCTATAGAGTTATATGATAAGATTGAGAAATTTAAAGTTGAT 1505**

**FJ173807： CTGATAAAGCTATAGAGTTATATGATAAGATTGAGAAATTTAAAGTTGAT 1505**

**FJ173813： CTGATAAAGCTATAGAGTTATATGATAAGATTGAGAAATTTAAAGTTGAT 1505**

**FJ173808： --------------------------------------------------**

**FJ173815： -------AGCTATAGAGTTATATGATAAGATTGAGAAATTTAAAGTTGAT 1654**

**FJ173805: TTTAAAAGCAGGCATGCCTGTGAATTAGGATGTATTTTATTGTTTATAAC　1555**

**EU375803： TTTAAAAGCAGGCATGCCTGTGAATTAGGATGTATTTTATTGTTTATAAC　1750**

**FJ173803： TTTAAAAGCAGGCATGCCTGTGAATTAGGATGTATTTTATTGTTTATAAC　1555**

**FJ173806： TTTAAAAGCAGGCATGCCTGTGAATTAGGATGTATTTTATTGTTTATAAC　1555**

**FJ173812： TTTAAAAGCAGGCATGCCTGTGAATTAGGATGTATTTTATTGTTTATAAC　1555**

**FJ173804： TTTAAAAGCAGGCATGCCTGTGAATTAGGATGTATTTTATTGTTTATAAC　1555**

**FJ173814： TTTAAAAGCAGGCATGCCTGTGAATTAGGATGTATTTTATTGTTTATAAC　1555**

**FJ173809： TTTAAAAGCAGGCATGCCTGTGAATTAGGATGTATTTTATTGTTTATAAC　1555**

**FJ173810： TTTAAAAGCAGGCATGCCTGTGAATTAGGATGTATTTTATTGTTTATAAC　1555**

**FJ173811： TTTAAAAGCAGGCATGCCTGTGAATTAGGATGTATTTTATTGTTTATAAC　1555**

**FJ173807： TTTAAAAGCAGGCATGCCTGTGAATTAGGATGTATTTTATTGTTTATAAC　1555**

**FJ173813： TTTAAAAGCAGGCATGCCTGTGAATTAGGATGTATTTTATTGTTTATAAC　1555**

**FJ173808： --------------------------------------------------**

**FJ173815： TTTAAAAGCAGGCATGCCTGTGAATTAGGATGTATTTTATTGTTTATAAC　1704**

**Forward primer**

**MCC1： GGCATGCCTGTGAATTAGGATGTATTTTATTGTTTATAAC　1750**

**MCC4： GGCATGCCTGTGAATTAGGATGTATTTTATTGTTTATAAC　1750**

**MCC6： GGCATGCCTGTGAATTAGGATGTATTTTATTGTTTATAAC　1750**

**MCC9： GGCATGCCTGTGAATTAGGATGTATTTTATTGTTTATAAC　1750**

**AK1： GGCATGCCTGTGAATTAGGATGTATTTTATTGTTTATAAC　1750**

**AK2： GGCATGCCTGTGAATTAGGATGTATTTTATTGTTTATAAC　1750**

**AK3： GGCATGCCTGTGAATTAGGATGTATTTTATTGTTTATAAC　1750**

**FJ173805: TTTATCAAAGCATAGAGTATTTGCTATTAAGAATTTTTGCTCTACCTTCT 1605**

**EU375803： TTTATCAAAGCATAGAGTATTTGCTATTAAGAATTTTTGCTCTACCTTCT 1800**

**FJ173803： TTTATCAAAGCATAGAGTATCTGCTATTAAGAATTTTTGCTCTACCTTCT 1605**

**FJ173806： TTTATCAAAGCATAGAGTATCTGCTATTAAGAATTTTTGCTCTACCTTCT 1605**

**FJ173812： TTTATCAAAGCATAGAGTATCTGCTATTAAGAATTTTTGCTCTACCTTCT 1605**

**FJ173804： TTTATCAAAGCATAGAGTATCTGCTATTAAGAATTTTTGCTCTACCTTCT 1605**

**FJ173814： TTTATCAAAGCATAGAGTATCTGCTATTAAGAATTTTTGCTCTACCTTCT 1605**

**FJ173809： TTTATCAAAGCATAGAGTATCTGCTATTAAGAATTTTTGCTCTACCTTCT 1605**

**FJ173810： TTTATCAAAGCATAGAGTATCTGCTATTAAGAATTTTTGCTCTACCTTCT 1605**

**FJ173811： TTTATCAAAGCATAGAGTATCTGCTATTAAGAATTTTTGCTCTACCTTCT 1605**

**FJ173807： TTTATCAAAGCATAGAGTATCTGCTATTAAGAATTTTTGCTCTACCTTTT 1605**

**FJ173813： TTTATCAAAGCATAGAGTATCTGCTATTAAGAATTTTTGCTCTACCTTCT 1605**

**FJ173808： --------------------------------------------------**

**FJ173815： TTTATCAAAGCATAGAGTATCTGCTATTAAGAATTTTTGCTCTACCTTCT 1754**

**MCC1： TTTATCAAAGCATAGAGTATCTGCTATTAAGAATTTTTGCTCTACCTTCT 1800**

**MCC4： TTTATCAAAGCATAGAGTATCTGCTATTAAGAATTTTTGCTCTACCTTCT 1800**

**MCC6： TTTATCAAAGCATAGAGTATCTGCTATTAAGAATTTTTGCTCTACCTTCT 1800**

**MCC9： TTTATCAAAGCATAGAGTATCTGCTATTAAGAATTTTTGCTCTACCTTCT 1800**

**AK1： TTTATCAAAGCATAGAGTATCTGCTATTAAGAATTTTTGCTCTACCTTCT 1800**

**AK2： TTTATCAAAGCATAGAGTATCTGCTATTAAGAATTTTTGCTCTACCTTCT 1800**

**AK3： TTTATCAAAGCATAGAGTATCTGCTATTAAGAATTTTTGCTCTACCTTCT 1800**

**FJ173805: GCACTATAAGCTTTTTAATTTGTAAAGGAGTGAATAAGATGCCTGAAATG　1655**

**EU375803： GCACTATAAGCTTTTTAATTTGTAAAGGAGTGAATAAGATGCCTGAAATG　1850**

**FJ173803： GCACTATAAGCTTTTTAATTTGTAAAGGAGTGAATAAGATGCCTGAAATG　1655**

**FJ173806： GCACTATAAGCTTTTTAATTTGTAAAGGAGTGAATAAGATGCCTGAAATG　1655**

**FJ173812： GCACTATAAGCTTTTTAATTTGTAAAGGAGTGAATAAGATGCCTGAAATG　1655**

**FJ173804： GCACTATAAGCTTTTTAATTTGTAAAGGAGTGAATAAGATGCCTGAAATG　1655**

**FJ173814： GCACTATAAGCTTTTTAATTTGTAAAGGAGTGAATAAGATGCCTGAAATG　1655**

**FJ173809： GCACTATAAGCTTTTTAATTTGTAAAGGAGTGAATAAGATGCCTGAAATG　1655**

**FJ173810： GCACTATAAGCTTTTTAATTTGTAAAGGAGTGAATAAGATGCCTGAAATG　1655**

**FJ173811： GCACTATAAGCTTTTTAATCTGTAAAGGAGTGAATAAGATGCCTGAAATG　1655**

**FJ173807： GCACTATAAGCTTTTTAATTTGTAAAGGAGTGAATAAGATGCCTGAAATG　1655**

**FJ173813： GCACTATAAGCTTTTTAATTTGTAAAGGAGTGAATAAGATGCCTGAAATG　1655**

**FJ173808： --------------------------------------------------**

**FJ173815： GCACTATAAGCTTTTTAATTTGTAAAGGAGTGAATAAGATGCCTGAAATG　1804**

**MCC1： GCACTATAAGCTTTTTAATCTGTAAAGGAGTGAATAAGATGCCTGAAATG　1850**

**MCC4： GCACTATAAGCTTTTTAATCTGTAAAGGAGTGAATAAGATGCCTGAAATG　1850**

**MCC6： GCACTATAAGCTTTTTAATCTGTAAAGGAGTGAATAAGATGCCTGAAATG　1850**

**MCC9： GCACTATAAGCTTTTTAATCTGTAAAGGAGTGAATAAGATGCCTGAAATG　1850**

**AK1： GCACTATAAGCTTTTTAATCTGTAAAGGAGTGAATAAGATGCCTGAAATG　1850**

**AK2： GCACTATAAGCTTTTTAATCTGTAAAGGAGTGAATAAGATGCCTGAAATG　1850**

**AK3： GCACTATAAGCTTTTTAATCTGTAAAGGAGTGAATAAGATGCCTGAAATG　1850**

**FJ173805: TATAATAATTTATGCAAGCCCCCTTACAAATTACTGCAAGAGAATAAGCC　1705**

**EU375803： TATAATAATTTATGCAAGCCCCCTTACAAATTACTGCAAGAGAATAAGCC　1900**

**FJ173803： TATAATAATTTATGCAAGCCCCCTTACAAATTACTGCAAGAGAATAAGCC　1705**

**FJ173806： TATAATAATTTATGCAAGCCCCCTTACAAATTACTGCAAGAGAATAAGCC　1705**

**FJ173812： TATAATAATTTATGCAAGCCCCCTTACAAATTACTGCAAGAGAATAAGCC　1705**

**FJ173804： TATAATAATTTATGCAAGCCCCCTTACAAATTACTGCAAGAGAATAAGCC　1705**

**FJ173814： TATAATAATTTATGCAAGCCCCCTTACAAATTACTGCAAGAGAATAAGCC　1705**

**FJ173809： TATAATAATTTATGTAAGTCCCCTTACAAATTACTGCAAGAGAATAAGCC　1705**

**FJ173810： TATAATAATTTATGTAAGTCCCCTTACAAATTACTGCAAGAGAATAAGCC　1705**

**FJ173811： TATAATAATTTATGTAAGCCCCCTTACAAATTACTGCAAGAGAATAAGCC　1705**

**FJ173807： TATAATAATTTATGCAAGCCCCCTTACAAATTACTGCAAGAGAATAAGCC　1705**

**FJ173813： TATAATAATTTATGCAAGCCCCCTTACAAATTACTGCAAGAGAATAAGCC　1705**

**FJ173808： --------------------------------------------------**

**FJ173815： TATAATAATTTATGCAAGCCCCCTTACAAATTACTGCAAGAGAATAAGCC　1854**

**Forward primer**

**MCC1： TATAATAATTTATGTAAGCCCCCTTACAAATTACTGCAAGAGAATAAGCC　1900**

**MCC4： TATAATAATTTATGTAAGCCCCCTTACAAATTACTGCAAGAGAATAAGCC　1900**

**MCC6： TATAATAATTTATGTAAGCCCCCTTACAAATTACTGCAAGAGAATAAGCC　1900**

**MCC9： TATAATAATTTATGTAAGCCCCCTTACAAATTACTGCAAGAGAATAAGCC　1900**

**AK1： TATAATAATTTATGTAAGCCCCCTTACAAATTACTGCAAGAGAATAAGCC　1900**

**AK2： TATAATAATTTATGTAAGCCCCCTTACAAATTACTGCAAGAGAATAAGCC　1900**

**AK3： TATAATAATTTATGTAAGCCCCCTTACAAATTACTGCAAGAGAATAAGCC　1900**

**Reverse primer**

**FJ173805: ACTGCTCAATTATGAATTTCAAGAAAAAGAAAAAGAGGCCAGCTGTAATT 1755**

**EU375803： ACTGCTCAATTATGAATTTCAAGAAAAAGAAAAAGAGGCCAGCTGTAATT 1950**

**FJ173803： ACTGCTCAATTATGAATTTCAAGAAAAAGAAAAAGAGGCCAGCTGTAATT 1755**

**FJ173806： ACTGCTCAATTATGAATTTCAAGAAAAAGAAAAAGAGGCCAGCTGCAATT 1755**

**FJ173812： ACTGCTAAATTATGAATTTCAAGAAAAAGAAAAAGAGGCCAGCTGTAATT 1755**

**FJ173804： ACTGCTCAATTATGAATTTCAAGAAAAAGAAAAAGAGGCCAGCTGTAATT 1755**

**FJ173814： ACTGCTCAATTATGAATTTCAAGAAAAAGAAAAAGAGGCCAGCTGTAATT 1755**

**FJ173809： ACTGCTCAATTATGAATTTTAAGAAAAAGAAAAAGAGGCCAGCTGTAATT 1755**

**FJ173810： ACTGCTCAATTATGAATTTTAAGAAAAAGAAAAAGAGGCCAGCTGTAATT 1755**

**FJ173811： ACTGCTCAATTATGAATTTCAAGAAAAAGAAAAAGAGGCCAGCTGTAATT 1755**

**FJ173807： ACTGCTCAATTATGAATTTTAAGAAAAAGAAAAAGAGGCCAGCTGTAATT 1755**

**FJ173813： ACTGCTCAATTATGAATTTTAAGAAAAAGAAAAAGAGGCCAGCTGTAATT 1755**

**FJ173808： --------------------------------------------------**

**FJ173815： ACTGCTCAATTATGAATTTCAAGAAAAAGAAAAAGAGGCCAGCTGTAATT 1904**

**MCC1: ACTGCTCAATTATGAATTTCAAGAAAAAGAAAAAGAGGCCAGCTGTAATT 1950**

**MCC4: ACTGCTCAATTATGAATTTCAAGAAAAAGAAAAAGAGGCCAGCTGTAATT 1950**

**MCC6: ACTGCTCAATTATGAATTTCAAGAAAAAGAAAAAGAGGCCAGCTGTAATT 1950**

**MCC9: ACTGCTCAATTATGAATTTCAAGAAAAAGAAAAAGAGGCCAGCTGTAATT 1950**

**AK1: ACTGCTCAATTATGAATTTCAAGAAAAAGAAAAAGAGGCCAGCTGTAATT 1950**

**AK2: ACTGCTCAATTATGAATTTCAAGAAAAAGAAAAAGAGGCCAGCTGTAATT 1950**

**AK3: ACTGC**

**FJ173805: GGAATTTAGTTGCTGAATTTGCTTGTGAATATGAGCTAGACGACCACTTT　1805**

**EU375803： GGAATTTAGTTGCTGAATTTGCTTGTGAATATGAGCTAGACGACCACTTT　2000**

**FJ173803： GGAATTTAGTTGCTGAATTTGCTTGTGAATATGAGCTAGACGA-------　1798**

**FJ173806： GGAATTTAGTTGCTGAATTTGCTTGTGAATATGAGCTAGACGACCACTTT　1805**

**FJ173812： GGAATTTAGTTGCTGAATTTGCTTGTGAATATGAGCTAGACGACCACTTT　1805**

**FJ173804： GGAATTTAGTTGCTGAATTTGCTTGTGAATATGAGCTAGACGACCACTTT　1805**

**FJ173814： GGAATTTAGTTGCTGAATTTGCTTGTGAATATGAGCTAGACGACCACTTT　1805**

**FJ173809： GGAATTTAGTTGCTGAATTTGCTTGTGAATATGAGCTAGACGACCACTTT　1805**

**FJ173810： GGAATTTAGTTGCTGAATTTGCTTGTGAATATGAGCTAGACGACCACTTT　1805**

**FJ173811： GGAATTTAGTTGCTGAATTTGCTTGTGAATATGAGCTAGACGACCACTTT　1805**

**FJ173807： GGAATTTAGTTGCTGAATTTGCTTGTGAATATGAGCTAGACGACCACTTT　1805**

**FJ173813： GGAATTTAGTTGCTGAATTTGCTTGTGAATATGAGCTAGACGACCACTTT　1805**

**FJ173808： --------------------------------------------------**

**FJ173815： GGAATTTAGTTGCTGAATTTGCTTGTGAATATGAGCTAGACGACCACTTT　1954**

**MCC1： GGAATTTAGTTGCTGAATTTGCTTGTGAATATGAGCTAGACGACCACTTT　2000**

**MCC4： GGAATTTAGTTGCTGAATTTGCTTGTGAATATGAGCTAGACGACCACTTT　2000**

**MCC6： GGAATTTAGTTGCTGAATTTGCTTGTGAATATGAGCTAGACGACCACTTT　2000**

**MCC9： GGAATTTAGTTGCTGAATTTGCTTGTGAATATGAGCTAGACGACCACTTT　2000**

**AK1： GGAATTTAGTTGCTGAATTTGCTTGTGAATATGAGCTAGACGACCACTTT　2000**

**AK2： GGAATTTAGTTGCTGAATTTGCTTGTGAATATGAGCTAGACGACCACTTT　2000**

**FJ173805: ATTATCTTAGCCCATTATCTAGACTTTGCAAAACCATTTCCTTGCCAAAA　1855**

**EU375803： ATTATCTTAGCCCATTATCTAGACTTTGCAAAACCATTTCCTTGCCAAAA　2050**

**FJ173803： ATTATCTTAGCCCATTATCTAGACTTTGCAAAACCATTTCCTTGCCAAAA　1855**

**FJ173806： --------------------------------------------------**

**FJ173812： ATTATCTTAGCCCATTATCTAGACTTTGCAAAACCATTTCCTTGCCAAAA　1855**

**FJ173804： ATTATCTTAGCCCATTATCTAGACTTTGCAAAACCATTTCCTTGCCAAAA　1855**

**FJ173814： ATTATCTTAGCCCATTATCTAGACTTTGCAAAACCATTTCCTTGCCAAAA　1855**

**FJ173809： ATTATCTTAGCCCATTATCTAGACTTTGCAAAACCATTTCCTTGCCAAAA　1855**

**FJ173810： ATTATCTTAGCCCATTATCTAGACTTTGCAAAACCATTTCCTTGCCAAAA　1855**

**FJ173811： ATTATCTTAGCCCATTATCTAGACTTTGCAAAACCATTTCCTTGCCAAAA　1855**

**FJ173807： ATTATCTTAGCCCATTATCTAGACTTTGCAAAACCATTTCCTTGCCAAAA　1855**

**FJ173813： ATTATCTTAGCCCATTATCTAGACTTTGCAAAACCATTTCCTTGCCAAAA　1855**

**FJ173808： --------------------------------------------------**

**FJ173815： ATTATCTTAGCCCATTATCTAGACTTTGCAAAACCATTTCCTTGCCAAAA　2004**

**MCC1： ATTATCTTAGCCCATTATCTAGACTTTGCAAAACCATTTCCTTGCCAAAA　2050**

**MCC4： ATTATCTTAGCCCATTATCTAGACTTTGCAAAACCATTTCCTTGCCAAAA　2050**

**MCC6： ATTATCTTAGCCCATTATCTAGACTTTGCAAAACCATTTCCTTGCCAAAA　2050**

**MCC9： ATTATCTTAGCCCATTATCTAGACTTTGCAAAACCATTTCCTTGCCAAAA　2050**

**AK1： ATTATCTTAGCCCATTATCTAGACTTTGCAAAACCATTTCCTTGCCAAAA　2050**

**AK2： ATTATCTTAGCCCATTATCTAGACTTTGCAAAACCATTTCCTTGCCAAAA　2050**

**Reverse primer**

**⇒　LT2 outer primer**

**FJ173805: GTGTGAAAACAGATCTCGCCTCAAACCTCACAAGGCTCATGAGGCTCATC　1905**

**EU375803： GTGTGAAAACAGATCTCGCCTCAAACCTCACAAGGCTCATGAGGCTCATC　2100**

**FJ173803： GTGTGAAAACAGATCTCGCCTCAAACCTCACAAGGCTCATGAGGCTCATC　1905**

**FJ173806： --------------------------------------------------**

**FJ173812： GTGTGAAAACAGATCTCGCCTCAAACCTCACAAGGCTCATGAGGCTCATC　1905**

**FJ173804： GTGTGAAAACAGATCTCGCCTCAAACCTCACAAGGCTCATGAGGCTCATC　1905**

**FJ173814： GTGTGAAAACAGATCTCGCCTCAAACCTCACAAGGCTCATGAGGCTCATC　1905**

**FJ173809： GTGTGAAAACAGATCTCGCCTCAAACCTCACAAGGCTCATGAGGCTCATC　1905**

**FJ173810： GTGTGAAAACAGATCTCGCCTCAAACCTCACAAGGCTCATGAGGCTCATC　1905**

**FJ173811： GTGTGAAAACAGATCTCGCCTCAAACCTCACAAGGCTCATGAGGCTCATC　1905**

**FJ173807： GTGTGAAAACAGATCTCGCCTCAAACCTCACAAGGCTCATGAGGCTCATC　1905**

**FJ173813： GTGTGAAAACAGATCTCGCCTCAAACCTCACAAGGCTCATGAGGCTCATC　1905**

**FJ173808： --------------------------------------------------**

**FJ173815： GTGTGAAAACAGATCTCGCCTCAAACCTCACAAGGCTCATGAGGCTCATC　2054**

**Forward primer**

**MCC1: GTGTG** **GATCTCGCCTCAAACCTCACAAGGCTCATGAGGCTCATC　2100**

**MCC4: GTGTG GATCTCGCCTCAAACCTCACAAGGCTCATGAGGCTCATC　2100**

**MCC6: GTGTG GATCTCGCCTCAAACCTCACAAGGCTCATGAGGCTCATC　2100**

**MCC9: GTGTG GATCTCGCCTCAAACCTCACAAGGCTCATGAGGCTCATC　2100**

**AK1: GTGTG GATCTCGCCTCAAACCTCACAAGGCTCATGAGGCTCATC　2100**

**AK2: GTGTG GATCTCGCCTCAAACCTCACAAGGCTCATGAGGCTCATC　2100**

**LT2 PCR**

**FJ173805: ATTCTAATGCTAAGCTATTTTATGAATCTAAATCTCAGAAAACCATTTGC　1955**

**EU375803： ATTCTAATGCTAAGCTATTTTATGAATCTAAATCTCAGAAAACCATTTGC　2150**

**FJ173803： ATTCTAATGCTAAGCTATTTTATGAATCTAAATCTCAGAAAACCATTTGC　1955**

**FJ173806： --------------------------------------------------**

**FJ173812： ATTCTAATGCTAAGCTATTTTATGAATCTAAATCTCAGAAAACCATTTGC　1955**

**FJ173804： ATTCTAATGCTAAGCTATTTTATGAATCTAAATCTCAGAAAACCATTTGC　1955**

**FJ173814： ATTCTAATGCTAAGCTATTTTATGAATCTAAATCT AGAAAACCATTTGC　1955**

**FJ173809： ATTCTAATGCTAAGCTATTTTATGAATCTAAATCT---AAGA--------　1944**

**FJ173810： ATTCTAATGCTAAGCTATTTTATGAATCTAAATCT---AAGACCATTTGC　1952**

**FJ173811： ATTCTAATGCTAAGCTATTTTATGAATCTAAATCTCAGAAAACCATTTGC　1955**

**FJ173807： ATTCTAATGCTAAGCTATTTTATGAATCTAAATCTCAGAAAACCATTTGC　1955**

**FJ173813： ATTCTAATGCTAAGCTATTTTATGAATCTAAATCTCAGAAAACCATTTGC　1955**

**FJ173808： --------------------------------------------------**

**FJ173815： ATTCTAATGCTAAGCTATTTTATGAATCTAAATCTCAGAAAACCATTTGC　2104**

**MCC1： ATTCTAATGCTAAGCTATTTTATGAATCTAAATCTCAGAAAACCATTTGC　2150**

**MCC4： ATTCTAATGCTAAGCTATTTTATGAATCTAAATCTCAGAAAACCATTTGC　2150**

**MCC6： ATTCTAATGCTAAGCTATTTTATGAATCTAAATCTCAGAAAACCATTTGC　2150**

**MCC9： ATTCTAATGCTAAGCTATTTTATGAATCTAAATCTCAGAAAACCATTTGC　2150**

**AK1： ATTCTAATGCTAAGCTATTTTATGAATCTAAATCTCAGAAAACCATTTGC　2150**

**AK2： ATTCTAATGCTAAGCTATTTTATGAATCTAAATCTCAGAAAACCATTTGC　2150**

**LT2 PCR**

**FJ173805: CAACAAGCCGCAGACACTGTTCTAGCCAAAAGGAGGTTAGAGATGCTGGA　2005**

**EU375803： CAACAAGCCGCAGACACTGTTCTAGCCAAAAGGAGGTTAGAGATGCTGGA　2200**

**FJ173803： CAACAAGCCGCAGACACTGTTCTAGCCAAAAGGAGGTTAGAGATGCTGGA　2005**

**FJ173806： ----------------------------------GGTTAGAGATGCTGGA　1814**

**FJ173812： CAACAAGCCGCAGACACTGTTCTAGCCAAAAGGAGGTTAGAGATGCTGGA　2005**

**FJ173804： CAACAAGCCGCAGACACTGTTCTAGCCAAAAGGAGGTTAGAGATGCTGGA　2005**

**FJ173814： CAACAAGCCGCAGACACTGTTCTAGCCAAAAGGAGGTTAGAGATGCTGGA　2005**

**FJ173809： --------------------------------------------------**

**FJ173810： CAGTTACC---------TATTTTCGCCAAA--------------------　1973**

**FJ173811： CAACAAGCCGCAGACACTGTTCTAGCCAAAAGGAGGTTAGAGATGCTGGA　2005**

**FJ173807： CAACAAGCCGCAGACACTGTTCTAGCCAAAAGGAGGTTAGAGATGCTGGA　2005**

**FJ173813： CAACAAGCCGCAGACACTGTTCTAGCCAAAAGGAGGTTAGAGATGCTGGA　2005**

**FJ173808： --------------------------------------------------**

**FJ173815： CAACAAGCCGCAGACACTGTTCTAGCCAAAAGGAGGTTAGAGATGCTGGA　2154**

**MCC1： CAACAAGCCGCAGACACTGTTCTAGCCAAAAGGAGGTTAGAGATGCTGGA　2200**

**MCC4: CAACAAGCCGCAGACACTGTTCTAGCCAAAAGGAGGTTAGAGATGCTGGA　2200**

**MCC6： CAACAAGCCGCAGACACTGTTCTAGCCAAAAGGAGGTTAGAGATGCTGGA　2200**

**MCC9： CAACAAGCCGCAGACACTGTTCTAGCCAAAAGGAGGTTAGAGATGCTGGA　2200**

**AK1： CAACAAGCCGCAGACACTGTTCTAGCCAAAAGGAGGTTAGAGATGCTGGA　2200**

**AK2： CAACAAGCCGCAGACACTGTTCTAGCCAAAAGGAGGTTAGAGATGCTGGA　2200**

**LT2 PCR**

**FJ173805: AATGACCAGGACAGAAATGCTATGTAAGAAGTTTAAGAAGCACCTAGAGA　2055**

**EU375803： AATGACCAGGACAGAAATGCTATGTAAGAAGTTTAAGAAGCACCTAGAGA　2250**

**FJ173803： AATGACCAGGACAGAAATGCTATGTAAGAAGTTTAAGAAGCACCTAGAGA　2055**

**FJ173806： AATGACCAGGACAGAAATGCTATGTAAGAAGTTTAAGAAGCACCTAGAGA　1864**

**FJ173812： AATGACCAGGACAGAAATGCTATGTAAGAAGTTTAAGAAGCACCTAGAGA　2055**

**FJ173804： AATGACCAGGACAGAAATGCTATGTAAGAAGTTTAAGAAGCACCTAGAGA　2055**

**FJ173814： AATGACCAGGACAGAAATGCTATGTAAGAAGTTTAAGAAGCACCTAGAGA　2055**

**FJ173809： --------------------------------------------------**

**FJ173810： ----------------------------------------CACCTC----　1979**

**FJ173811： AATGACCAGGACAGAAATGCTCTGTAAGAAGTTTAAGAAGCACCTAGAGA　2055**

**FJ173807： AATGACCAGGACAGAAATGCTATGTAAGAAGTTTAAGAAGCACCTAGAGA　2055**

**FJ173813： AATGACCAGGACAGAAATGCTATGTAAGAAGTTTAAGAAGCACCTAGAGA　2055**

**FJ173808： --------------------------------------------------**

**FJ173815： AATGACCAGGACAGAAATGCTATGTAAGAAGTTTAAGAAGCACCTAGAGA　2204**

**MCC1： AATGACCAGG**

**MCC4: AATGACCAGG**

**MCC6： AATGACCAGG**

**MCC9： AATGACCAGG**

**AK1： AATGACCAGG**

**AK2： AATGACCAGG**

**Reverse primer**

**LT2 PCR**
